# High-Resolution Mass Spectrometry Method for Targeted Screening and Monitoring of Fabry, Gaucher and ASMD Using Dried Blood Spots and Capitainers: Impact of Sample Matrix on Measurement Results

**DOI:** 10.3390/ijms26157641

**Published:** 2025-08-07

**Authors:** Amber Van Baelen, Stijn Verhulst, François Eyskens

**Affiliations:** 1 Center of Inherited Metabolic Diseases, University Hospital of Antwerp, 2650 Antwerp, Belgium; francois.eyskens@uza.be; 2 Laboratory of Experimental Medicine and Pediatrics, University of Antwerp, 2610 Antwerp, Belgium; stijn.verhulst@uza.be; 3 Pediatric Department, University Hospital of Antwerp, Drie Eikenstraat 655, 2650 Edegem, Belgium; 4 Metabolic Lab Chromatography, University Hospital of Antwerp, 2650 Antwerp, Belgium

**Keywords:** Lysosomal storage disease, sphingolipidoses, Gaucher, Fabry, ASMD, Lyso-Gb1, GlcSph, Lyso-Gb3, Lyso-SM, biomarker, dried blot spot, capitainer, filter system, LC-MS/MS, screening

## Abstract

The sphingolipidoses Fabry disease, Gaucher disease and Acid sphingomyelinase deficiency (ASMD) are the three most common lysosomal storage diseases for which treatment is currently available. Timely diagnosis with estimation of the disease severity and possibilities of follow-up of patients, whether or not under therapy, is crucial for providing good care and for the prevention of possible lethal complications. With this research we provide an efficient and sensitive detection method; its implementation in clinical practice could optimize the diagnosis and follow-up of patients with Gaucher, Fabry and ASMD. This detection method on dried blood spots (DBS) was validated according to the international Clinical and Laboratory Standards Institute (CLSI) guidelines, looking at reproducibility, linearity, carry-over and lower limit of quantification. Analogously, validation and subsequent comparison of the method validation results using another matrix, the Capitainer blood sampling cards (Capitainers), was fulfilled. The results showed that this detection method is fully applicable clinically when using DBS as well as Capitainers. In addition, even additional improvements of some validation parameters were found when using the Capitainers. Twenty-six patient samples and fifteen healthy samples were analyzed for case finding control. All patient cases were detected without ambiguity. We present a high-resolution mass spectrometry method that provides an accurate analysis for targeted screening, aiming for improved/accelerated diagnosis when added in the diagnostic pathway and monitoring of Fabry, Gaucher and ASMD in DBS as well as in Capitainers, with the main advantages of a small volume of blood samples, guaranteeing stability and easy transportation from the collection site to the laboratory.

## 1. Introduction

The search for detectable biomarkers to support the diagnostic search for a wide spectrum of disorders is a clinically impactful field of research. When sensitive and specific biomarkers are found, with usefulness in patient care, the next phase is to develop a detection method to implement them in clinical practice.

Lyso-glucosylsphingosine (GlcSph), Lyso-globotriaosylsphingosine (Lyso-Gb3), and Lyso-sphingomyelin (Lyso-SM) are three of these biomarkers found to be very sensitive and specific for diagnosing their associated disorder resp. Gaucher disease (GD), Fabry disease (FD) and acid sphingomyelinase deficiency (ASMD) [1,2,3,4,5,6,7]. All three disorders belong to the group of Spingolipidoses, a part of the Lysosomal storage disorders (LSDs), which encompass a diverse group of inherited metabolic diseases caused by deficiencies in specific lysosomal enzymes due to specific genetic mutations [8].

**Figure 1:**
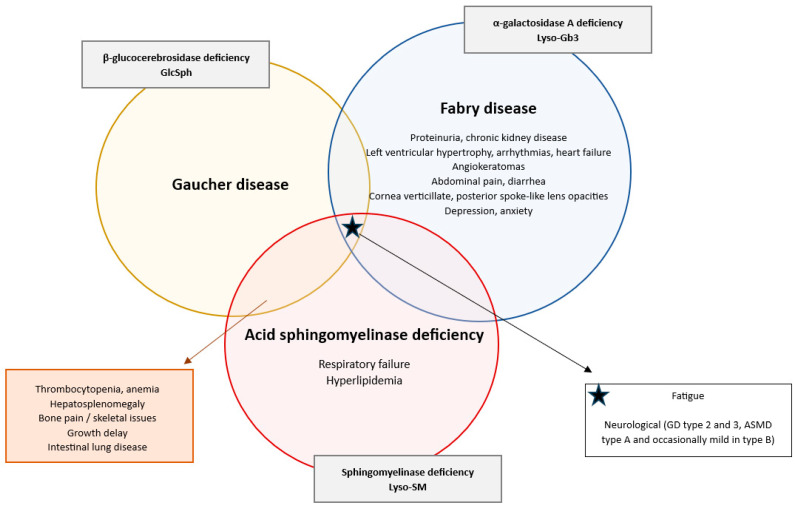
Diagram depicting the clinical symptomatology of Gaucher disease, Fabry disease and ASMD: shared and distinct features in lysosomal storage disorders. This diagram contains the typical clinical symptoms of Gaucher disease, Fabry disease and ASMD [9,10,15,16,17,19,20,21,22]. Disease-specific symptoms that are unique to one of the three disorders are noted inside the corresponding circle. Overlapping clinical symptoms between two disorders are listed in the boxes connected with an arrow to the relevant overlapping sections. The starred box lists the symptoms common to all three disorders. The gray boxes indicate the specific enzyme deficiency and associated lysosphingolipid biomarker for each specific disorder.

Excess Lyso-glucosylsphingosine (GlcSph) concentration develops due to the dysfunction of the enzyme β-glucocerebrosidase, resulting in the accumulation of glucosylceramide (Gb-1), which gets deacylated to GlcSph in patients with GD [1,2,3]. The enzyme dysfunction is caused by the *GBA1 gene* mutation on chromosome 1 (1q21) [9]. Its prevalence varies widely, from approximately 0.4 to 5.8 per 100,000 individuals in general, but it is significantly higher in specific ethnic groups, such as the Ashkenazi Jewish population, where the incidence is about 1 in 800 individuals [3]. The accumulation of Gb1 predominantly affects the liver, spleen, and bone marrow, resulting in a range of clinical symptoms, hallmarked by hepatosplenomegaly and thrombocytopenia (Figure 1) [9,10].

Similarly, Lyso-globotriaosylsphingosine (Lyso-Gb3) results from a deficiency in α-galactosidase A, causing an increase in globotriaosylceramide (Gb3), which gets deacylated into Lyso-Gb3 in patients with FD [4,5]. The genetic mutation causing the enzyme deficiency is a mutation in the *GLA gene* on the X chromosome [11]. The incidence of FD is estimated to be around 1 in 8454 to 117,000 male births [12,13]. Since it is an X-linked disorder, males typically experience more severe symptoms than female patients [13,14]. The lysosomal accumulation affects cardiovascular, renal, neurological, and dermatological systems. Symptoms can include acroparesthesias, hypohidrosis, angiokeratomas, cardiomyopathy, proteinuria, renal failure, cryptogenic stroke and ocular issues, e.g., cornea verticillate (Figure 1) [15,16].

Lastly Lyso-sphingomyelin (Lyso-SM) gets increased due to the deficiency of acid sphingomyelinase, causing an accumulation of sphingomyelin, which gets deacylated into Lyso-SM [6,7]. The mutation responsible for ASMD is a mutation in the *sphingomyelin-phosphodiesterase 1 gene (SMPD1)* located on chromosome 11 (11p15.4) [17]. The estimated incidence of ASMD is 0.4–0.6 in 100,000 individuals [18]. ASMD presents a clinical profile that closely resembles that of GD, particularly with features such as hepatosplenomegaly as one of the hallmarks of both disorders, as well as thrombocytopenia, coagulopathy, bone involvement with growth delay, bone pain and osteopenia/osteoporosis, hyperlipidemia and pulmonary complications, the latter more present in ASMD patients than in GD patients (Figure 1) [17,19,20,21,22]. The diverse manifestations and rarity of ASMD often lead to diagnostic delays, particularly when other urgent hematological conditions are considered [6,23,24].

For all three LSD counts, due to their rarity and very diverse and clinical presentations, they are often underdiagnosed or misdiagnosed, especially in the context of other urgent hematological, less rare, conditions [14,25]. Since accurate diagnosis and effective monitoring of these conditions are crucial for timely intervention and improved patient outcomes, the importance of good diagnostic tests along with robust reference ranges from large healthy cohorts raises and emphasizes the need for reliable biomarkers such as GlcSph, Lyso-Gb3 and Lyso-SM added to the standard diagnostic pathway based on enzymatic and genetic screening.

GlcSph, Lyso-Gb3 and Lyso-SM have not only been shown to enhance diagnostic accuracy but also facilitate monitoring of disease progression and treatment efficacy [26,27,28,29]. Additionally, higher biomarker concentrations have been correlated with more severe phenotypes of the corresponding disorder, making estimation of further clinical expectations more feasible [27,30,31]. While enzyme assays and genetic testing can only give you information about a possible diagnosis, these biomarkers go far beyond and help the clinician make more informed decisions in their treatment options [6].

In case of lysosomal biomarker analyzations, capillary blood sampling remains a cornerstone due to its minimally invasive and low-volume collection. Dried blood spots (DBSs) are a matrix using filter paper to spot whole blood and then transport and store it [1,2,5,32]. It is a medium that has been widely demonstrated to be stable and easy to use with numerous advantages in terms of storage, transport, and finances [5,33,34]. However, the traditional DBS collection is susceptible to issues such as hematocrit variability and inconsistent sample volumes, which might influence the analytical measurement. Recent advancements in the volumetric absorptive microsampling devices (VAMSs) delivered an additional new matrix, the Capitainer blood sampling cards (Capitainer^®^qDBS, CAP) developed by Capitainer AB [35]. This matrix is a more sophisticated dried blood spot that provides an answer to the standard DBS limitations by ensuring volumetric precision and improved standardization with its microfluidic internal filter mechanism to control volume and ensure that exactly 10 µL of blood is collected on each spot, regardless of the hematocrit [35]. In the analytical context of lysosomal storage disorders, the incorporation of CAP seems not intended to replace DBS but rather to enhance it, offering a more robust alternative sample matrix in cases where volume and hematocrit might play an influential role.

This article describes an extension and improvement of the detection method already published that utilizes DBS samples for the simultaneous measurement of these three biomarkers, aiming to streamline the diagnostic process and improve patient care [1,3,33,36]. Adding the use of CAP brings innovation in the use of these diagnostic tests for LSDs. The use of DBS and CAP allows for easier sample collection and preservation during transport and storage, resulting in fewer issues with the samples occurring and thereby potentially increasing lab efficiency and accelerating the initiation of therapy [1,3,33].

## 2. Results

### 2.1. The LC-MS/MS Method

Building on the previously developed method for two biomarkers, preliminary experiments were performed to incorporate the third biomarker, Lyso-SM, into the detection technique [36]. Experiments to optimize the spectrometric parameters and mobile phases were performed. Direct infusion of Lyso-SM d7 and Lyso-SM 10 ppm solutions was used to define the ion source parameters in positive ion mode.

### 2.2. Analytical Validation

**Figure 2:**
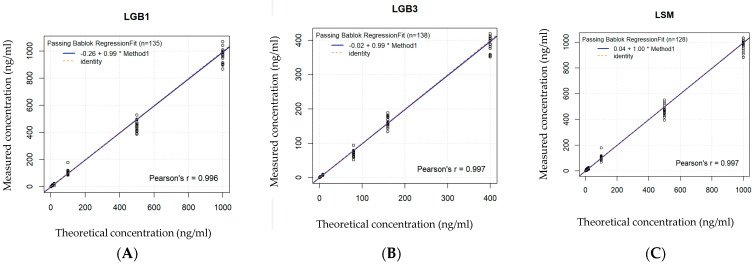
(**A**–**C**): Linearity of the calibration curve in DBS. Based on 7 standard values, linearity is shown for each biomarker by comparing the spiked concentrations with the effectively measured concentrations. Concentration units are expressed in ng/mL (or 10^−6^ g/L). The * stands for the x in the regression equation, see Table 1. (**A**) Bland–Altman curve for GlcSph. (**B**) Bland–Altman curve for Lyso-Gb3. (**C**) Bland–Altman curve for Lyso-SM.

**Figure 3:**
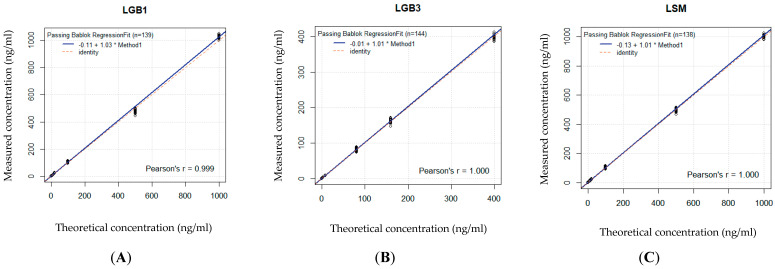
(**A**–**C**): Linearity of the calibration curve in CAP. Based on 7 standard values, linearity is shown for each biomarker by comparing the spiked concentrations with the effectively measured concentrations. Concentration units are expressed in ng/mL (or 10^−6^ g/L). The * stands for the x in the regression equation, see Table 1. (**A**) Bland–Altman curve for GlcSph. (**B**) Bland–Altman curve for Lyso-Gb3. (**C**) Bland–Altman curve for Lyso-SM.

A good significant correlation between the measured and the theoretical concentrations proved linearity of the calibration curve in DBS as well as in CAP for GlcSph, Lyso-Gb3 and Lyso-SM based on seven different standard concentrations (Figure 2A–C and Figure 3A–C and Table 1).

Based on the defined QC values, the measurements for inter-assay and intra-assay accuracy and precision were represented in Table 2 and Table 3. For all DBS levels a value was achieved below the predefined cut-off of 15% except for the two lowest QC levels of Lyso-SM for precision and the second level for accuracy and QC 1, 2 for accuracy for Lyso-Gb3. For the CAP all levels were achieved below the predefined cut-off of 15% except for the lowest intra-run precision for Lyso-SM.

Low after low runs versus the low after high runs differences in absolute numbers were 0.71 for GlcSph, 0.29 for Lyso-Gb3 and 1.92 for Lyso-SM. All values fall significantly below the established threshold, indicating that the method is free from any carry-over (Table 4).

The S/N of all three biomarkers, as well as in DBS and CAP, was found to be clearly above 10 for LOQ (Table 5).

### 2.3. Case Finding Results

Measurements for the concentration of each biomarker in the included samples are shown in Table 6. Comparison between the healthy control samples (N = 15) and disease samples (N = 26) was performed by using the Mann–Whitney U test, since the Shapiro–Wilk test showed that, in contrast to the healthy samples, the diseased data for all three analyses were not normally distributed. Mann–Whitney U test showed a significant difference for Lyso-Gb3 when comparing healthy versus disease samples, *p*-value = 0.000023 (****). There was no significant difference between healthy and disease samples for GlcSph and Lyso-SM, which can be explained by the great inter-individual variance in the diseased group, since it consists of three subgroups, namely, 9 Gaucher samples, 7 Fabry samples and 10 ASMD samples.

When looking at the differences between the different disease groups, namely, Gaucher versus Fabry versus ASMD, using the Kruskal–Wallis test, significant differences were shown in all three analyte groups: GlcSph (*p*-value = 0.000022 (****)), Lyso-Gb3 (*p*-value = 0.0000061 (****)) and Lyso-SM (*p*-value = 0.000013 (****)).

**Figure 4:**
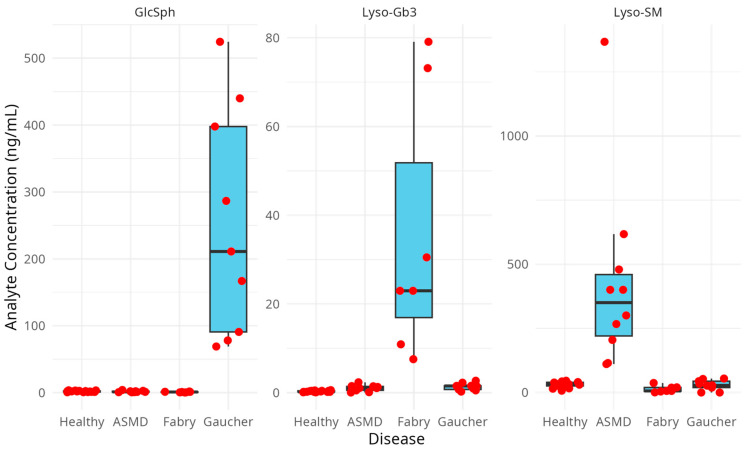
Boxplot representing the biomarker levels in each sample group. The red dots are the individual measurement results, which can be found in Table 6.

The Mann–Whitney U test was used as a post hoc pairwise differentiation test, which showed that the values of each specific biomarker level were significantly different between the related specific disease samples (Gaucher for GlcSph, Fabry for Lyso-Gb3 and ASMD for Lyso-SM) compared with the healthy controls. GlcSph levels in Gaucher patients versus healthy controls were as follows: *p*-value = 0.0000015 (****); Lyso-Gb3 levels in Fabry patients versus healthy controls were as follows: *p*-value 0.00024 (***) and Lyso-SM levels in ASMD patients versus healthy controls were as follows: *p*-value 0.000036 (***). Additionally there was a significant difference for GlcSph values between Fabry patients and healthy controls (*p*-value = 0.016 (*)), for Lyso-Gb3 values between ASMD patients and healthy controls (*p*-value = 0.013 (*)), for Lyso-Gb3 values between Gaucher patients and healthy controls (*p*-value = 0.00043 (***)) and for Lyso-SM values between Fabry patients and healthy controls (*p*-value = 0.011 (*)). The patient samples, analyzed with this method, showed a clear distinction between the healthy and patient samples as well as between different types of lysosomal disorders (Figure 4).

## 3. Discussion

By enabling simultaneous measurement of GlcSph, Lyso-Gb3, and Lyso-SM in both biological matrices, clinicians can enhance diagnostic accuracy since these biomarkers can inherently differentiate between LSDs, especially when combined with clinical examination and enzymatic analyzation, and thereby consequently improve patient outcomes by early treatment and closely monitoring efficacy of this treatment. This expansion on the previously published detection method on DBS samples as well as the addition of the Capitainers, a new matrix, offers a reliable option to achieve these advancements in the diagnosis and management of sphingolipidoses/lysosomal storage diseases [36].

More specifically, this combination method is of great interest in clinical presentations with, for example, splenomegaly and possibly additional thrombocytopenia, which are the characteristic features of both Gaucher disease and ASMD as explained in the Introduction and confirmed in our own experience [17,19,20,21,22,37]. Due to the impossibility of clinically differentiating between both conditions, this combination test offers a simplified differentiation where the same minimal material (at least 1 circle (diameter 13 mm) on a heel prick card, 70 µL of blood or 1 Capitainer spot, 10 µL) is needed to be able to establish this. However effective the ability to differentiate between GD and ASMD, the research pointed out that distinguishing the isomeric compounds hexosphingosine, GlcSph and psychosine (galactosylsphingosine) is a challenge. However, given the clinical/phenotypic distinctions between Gaucher and Krabbe diseases added to the available specific enzyme assays, misdiagnosis will not be an issue, and isomeric separation is not necessary [3,38].

Additionally the method can offer diagnostic support in the diagnosis of Fabry patients where the diagnostic process first-tier is established in males by alpha-GAL A enzymatic activity in leucocytes and by gene sequencing in females [39]. This biomarker detection method can be very complementary in female patients as well as patients with the attenuated phenotype where the enzymatic activity is often within the normal ranges [38]. A recent study also suggests that the ratio of α-gal A activity to Lyso-Gb3 in dried blood spots may serve as a valuable diagnostic marker in females. Lyso-Gb3 is not specific for Fabry disease and has been reported in patients with mucopolysaccharidoses (MPS 1, MPS 2 and MPS 3) although the phenotypic presentation of these disorders is completely different from Fabry disease [40].

Finally interesting correlations between these biomarkers and the clinical severity or their correlated LSDs have been pointed out [6,27,30,31,41]. They have been used as a monitoring biomarker for enzyme replacement therapy as well as a surrogate biomarker in clinical studies and for clinical management [26,29,42]. While enzyme assays and genetic testing can only give you information about a possible diagnosis, these biomarkers go far beyond and help the clinician make more informed decisions in their treatment options, pointing out their importance and clinical potential [6].

Compared to other possible sampling methods, the use of capillary blood sampling options excels in their minimally invasive, low-volume collection compared with, for example, alternative sampling methods using standard phlebotomy or vacutainer tubes [43]. When comparing DBS with CAP samplings, the advantages of DBS are its reduced costs and user-friendly experience based on its widespread implementation and familiarity in clinical settings. Additionally the use of DBS aligns with the current medium used for enzymatic analysis and newborn screenings. This ensures compatibility should the test be integrated into future screening programs, whereas alternative sampling methods are not suitable for analysis on the same specimen type.

The validation results in this research demonstrate that both the DBS detection method as well as the CAP detection method are ready and valuable to implement in clinical practice. Comparing the validation results for both matrices, we can conclude that the CAP adds value in the detection of the three biomarkers with special specific interest in Lyso-SM. The only limitation observed for CAP was a slight increase above the predetermined CLSI cut-off in intra-assay precision for Lyso-SM and Lyso-Gb3. The observed lower precision for the lowest QC concentration of Lyso-Gb3 when using CAP is due to a detectable noise specific to the CAP matrix that interferes with the measurement of the lowest QC level; however, when using the DBS samples, the results meet all validation conditions for the method with each determination well under the proposed cut-off of 15%. For Lyso-SM, the concentration of this lowest QC level at which the variability was detected is not clinically relevant. This is because the threshold for distinguishing a patient sample with ASMD from a healthy sample is 20 to 50-fold higher than the concentration of the lowest QC level used in the validation process. This points out that the method has no relevance for clinical interpretation in this low concentration zone. In clinical practice the second or even the third level QC is more appropriate as the first concentration for quality control purposes. For all other and clinically relevant concentrations, the results were well below the predetermined cut-off of 15% variability determined by the international CLSI guidelines. This indicates that the CAP is highly effective for determining the three biomarkers, especially for Lyso-SM levels when comparing with the DBS results.

Additionally a difference can be noted in the accuracy of the lower levels comparing DBS to CAP results, where we notice some elevations in the lowest QC levels of the DBS validation results and fulfilled criteria for the CAP validation results. This difference and improvement by using CAP can be explained by the filter system that lies at the base of the CAP, resulting in less matrix influence based on the analyzed blood concentration represented in the difference in accuracy between both matrices. However, the elevated accuracy for the lowest levels of Lyso-Gb3 on DBS is rather relative since the absolute numbers used for the calculation do not differ by more than 0.5 from the intended target in absolute numbers as well as the mean (resp. QC1 mean 1.5 ng/mL and QC2 mean 3.75 ng/mL) and SD values (resp. QC1 SD withing 0.19, SD between 0.14 and QC2 SD within 0.3, SD between 0.51).

Both matrices fulfilled all the requirements for obtaining a significant linear correlation, absence of carry-over and satisfying LOQ results.

Given the above-described advantages and limitations of each DBS and CAP, we can conclude that, for the determination of Lyso-Gb3 in the follow-up and diagnosis of Fabry disease, the detection by using DBS cards is preferred. In contrast for the follow-up of patients with ASMD analysis there is a preference for using CAP in order to improve sensitivity and specificity in the detection of Lyso-SM.

When implementing this combination method, several other advantages emerge for future clinical practices. It is both time saving and cost-effective compared to alternative detection methods or alternative ways (such as genetic diagnostics). Moreover it allows for easy and reliable transport of samples over long distances, which is particularly beneficial for patients with mobility restrictions, and follow-up can be sent from home.

Looking at real-world data, the case findings showed a complete detection coverage with a clear distinction between healthy (all biomarkers have normal levels) and affected patients (depending on the LSD, the specific correlated biomarker is elevated). Based on these results, we can conclude that not only the statistical validation of the method but also the clinical interpretation is on point when using this method on real-world patient samples. However, we used all confirmed patient samples available in our center, which was already a remarkable amount because of the rarity of the disorders. Expanding the sample size further would allow for even more robust and conclusive findings. In order to further scientifically substantiate the assessment of samples, additional research is currently being conducted to define reference values on a large scale of patient samples.

Last but not least, when extending the high accuracy, sensitivity and specificity of the method based on the case finding results, we can point out that our method of analysis of Lyso-biomarkers on dried blood spots yield great promise towards the implementation of early detection of sphingolipidoses in newborn screening as second-tier test following enzymatic assay or genetic first analysis as a first-tier screening test [44,45].

## 4. Materials and Methods

### 4.1. Chemicals and Reagents

Globotriaosylsphingosine (Lyso-Gb3), Lyso-glucosylsphingosine (GlsSph) and Sphingosylphosphorylcholine (Lyso-SM) (all with a purity ≥ 98 and, respectively, molecular weights of 786 g/mol, 462 g/mol and 464.6 g/mol) were purchased from Matreya LLC, State College, PA, USA, and dissolved in Chloroform/Methanol (2:1) to make a 1 mg/mL stock solution.

Sphingosylphosphorylcholine-d7 (Lyso-SM-d7) (Purity ≥ 98%, molecular weight X g/mol) was used as internal standard and purchased from Avanti Polar Lipids, INC, USA. ^13^C_6_-Globotriaosylsphingosine (^13^C_6_-Lyso-Gb3) (purity ≥ 98%, molecular weight 791.87 g/mol) was used as internal standard and purchased from GelbChem, Seattle WA, USA. ^13^C_6_-Glucosylsphingosine (^13^C_6_-GlcSph) (purity ≥ 98%, molecular weight X g/mol) was also used as internal standard and purchased from Cayman Chemical Company, Ann Arbor, MI, USA.

Analytical chemicals and solvents include Formic acid (Purity 99–100%), purchased from VWR Chemicals, France. Acetonitrile UPLC, Water ULC/MS (Purity ≥ 99%), Methanol ULC/MS-CC/SFC (MeOH, Purity 99.98%), and Isopropranol UPLC (Purity ≥ 99%) were all purchased from Biosolve chimie SARL, Dieuze, France. Dimethylsulfoxide (DMSO, Purity 99.9%) and Chloroform (Purity 99–99.4%) were purchased from Merck, Sigma Aldrich, Darmstadt, Germany. Physiological water (Purity ≥ 99%) was purchased from Baxter, Switzerland.

### 4.2. Standards, Internal Standards and Quality Controls

Standards, internal standards and quality controls were defined and made analogous to previously published research [36]. Based on the CLSI C62 and EP05 guidelines, the validation protocol was defined. Additional information compared to previous publication that should be noted is the preparation of the standards and quality controls for Lyso-SM. Concentrations were, based on reported ranges from healthy individuals and patients with ASMD in research, defined as analogous to those for GlcSph. This means that serial dilution of the stock solution with DMSO/MeOH (1:1) at levels of 100, 500, 1000, 2000, 10,000, 50,000 and 100,000 ng/mL was used to prepare the standards. A combination of the three biomarkers was prepared by adding 10 µL of the different levels and afterwards dissolved with a 1:1 ratio of washed red blood cells (RBCs) and DMSO/physiological water (52%:48%), making a 100 times dilution and resulting in the final concentrations of 1, 5, 10, 20, 100, 500 and 1000 ng/mL for GlcSph and Lyso-SM and 0.2, 0.5, 2, 8, 80, 160 and 400 ng/mL for Lyso-Gb3, which defined the calibration standards (Table 7). Analogous Quality Controls (QCs) were made from their specific stock solutions (1 mg/mL), finishing with the concentrations of 400, 1000, 4000, 20,000 and 80,000 ng/mL for Lyso-SM, followed by dilution with washed RBC, DMSO and physiological water. The prepared QC concentrations were 4, 10, 40, 200 and 800 ng/mL for GlcSph and Lyso-SM and 1.2, 3, 10, 50, 200 ng/mL for Lyso-Gb3, providing values close to the LOQ, medium and high levels (Table 8). Preparation of the levels spotted onto filter paper (PerkinElmer, Turku, Finland) and the Capitainer qDBS (Capitainer AB, Solna, Sweden) was identical to the referenced paper. All samples were stored at −20 °C in zip-lock plastic bags with desiccant until further analysis.

### 4.3. Sample Preparation

The sample preparation was described with much detail in a recent publication by this research group (bron). During the process some small adjustments to improve the sensitivity and accuracy of the method were implemented. To improve the detection sensitivity, not only was ^13^C_6_-Lyso-Gb3 dissolved (2 µL/ ml) to the 150 µL extraction solution (DMSO/MeOH; 1:1) but also ^13^C_6_-Lyso-Gb1 and Lyso-SM d7 were added as well. Extraction was obtained by sonication for 30 min at 30 °C and shaking for 20 min at 37 °C as incubation at 300 rpm (PerkinElmer, TriNEST TM incubator shaker). To obtain a clear supernatant, centrifugation (Beckman Coulter, Allegra x-15R) for 10 min at 4750 rpm and 20 °C was followed by separating 100 µL of the supernatant layer into a 96-well microplate (Waters, Milford, MA, USA). To ensure that there would be no material causing any obstruction on the LC-MSMS, the samples were centrifuged for 10 min for additional purification. Five µL was injected into the liquid chromatography tandem mass spectrometry (LC-MS/MS) system.

Calibration curves were established for each biomarker across all matrices to ensure accurate quantification. Quality control samples were included in each run to monitor analytical performance, and intra- and inter-assay variability were evaluated to assess method robustness.

### 4.4. Liquid Chromatography with Tandem Mass Spectrometry

The liquid chromatography tandem mass spectrometry (LC-MS/MS) system used was a QTRAP5500 (AB Sciex, Framingham, MA, USA) detector with Nexera X2 LC-30AD ultra-high performance liquid chromatography pumps (Shimadzu Scientific Instruments, Columbia, MD, USA). Specific settings for the QTRAP5500 system are analogous to previously published research [36] and are provided in Table 9.

The Multiple Reaction Monitoring (MRM) transitions for ^13^C_6_-Lyso-Gb3, ^13^C_6_-Lyso-Gb1, Lyso-SM and Lyso-SM d7 were, respectively, 792.392 > 282.3 *m*/*z*, 469.300 > 282.300 *m*/*z*, 465.300 > 184.00 *m*/*z* and 472.500 > 184.100 *m*/*z*.

A C18 column (Acquity UPLC CSH C18 1.7 µm, 2.1 mm × 50 mm, Waters, Milford, MA, USA) with 40 °C as column temperature was used for sample separation. A precolumn (Waters, USA) was added to protect the column from contamination.

A gradient elution, with a flow rate of 0.05 mL/min, was made with 0.1% formic acid in water as solvent A and 0.1% formic acid in 80% acetonitrile and 20% MeOH as solvent B. The gradient started at 75%A–25%B, gradually changed to 0%A-100%B at 2.5 min, and returned to the initial conditions after 0.1 min. The total run time was 5 min in total. The washing solution was 30%MeOH:30%Water:30%Acetonitrile:10%Isopropranol.

Retention time was set at 1.86 min for GlcSph, 1.77 min for Lyso-Gb3 and 1.78 min for Lyso-SM.

### 4.5. Method Validation

The developed method, reported in Table 10, was validated according to the official CLSI guidelines (CLSI C50, C62, E17, EP 06, EP05 and EP10). The CLSI guidelines are widely recognized guidelines and provide a comprehensive framework specifically tailored for the validation of clinical laboratory methods. The specific CLSI guidelines, as listed, were selected based on their specific topics, including linearity, accuracy, precision, and carry-over analyses. These guidelines advised using 20 individual runs with 3 series of the predetermined samples for the validation calculations, resulting in 60 analytical results to define the between- and within-run accuracy and precision, carry-over and limit of quantification (LOQ). R statistical software v2.10.1 (Revolution Analytics, Palo Alto, CA, USA) was used for completing statistical analyzations.

#### 4.5.1. Linearity

Based on 7 calibration standards, linearity of the method was defined in the 20 different runs. A linear calibration curve was determined by the ratio of the peak area of the detected versus the spiked concentrations. Using a 1/x weighing the correlation between the measured results and the target values needs to be below 15% (CLSI EP06). Tests used for the calculations were Passing Bablok regression, Spearman’s correlation, and the Bland–Altman plot.

#### 4.5.2. Precision

Triplet analyzation of the quality control levels for each individual run resulted in precision (CV%) calculations for the intra-day test. Analogously the inter-day test (100 × (the standard deviation/calculated mean)) was carried out by analyzing the levels in total. Comparison of the measured and spiked concentrations determined the precision. As defined in the CLSI guidelines (C62 and EP05), CV% is required to be ≤15%. The chi-quadrate test was used to calculate if the obtained results were below the predetermined cut-off. Differences with *p* < 0.05 were significant.

#### 4.5.3. Accuracy

Triplet analyzation of the quality control levels for each individual run ((calculated mean − nominal value)/nominal value × 100) and in 20 replicates for inter-day test determined the accuracy (bias%). Comparison of the measured and spiked concentrations determined the accuracy, as defined in the CLSI guidelines (C62 and EP05). The chi-quadrate test was used to calculate if the obtained results were below the predetermined cut-off. Differences with *p* < 0.05 were significant.

#### 4.5.4. Carry-Over

By analyzing the variation between 6 low levels after low levels and 5 low levels analyzed after high levels, a carry-over analysis was performed according to the CLSI EP10 guidelines. The difference between the mean detected concentrations of both series defined the calculated value for carry-over. The methodology is free from any carry-over if the calculated value is less than 3 times the standard deviation of the lowest value.

#### 4.5.5. Lower Limit of Quantification

The signal-to-noise method, CLSI guidelines E17 and C50, were used to define the limit of quantification (LOQ). The auto-integrator of the instrument can be used to calculate the LOQ, or a manual calculation on a chromatogram printout can be used. The signal-to-noise ratio (S/N), defined as the ratio of the peak signal to the noise signal, should exceed 10. To account for potential variation between different runs, the LOQ was calculated across 5 separate measurements for each biomarker. The final LOQ value was determined by using the mean of these runs. The area used for calculating the LOQ is defined by the full width of the peak, from the starting point to the baseline endpoint.

#### 4.5.6. Case Finding

A collection of 26 patient samples (9 Gaucher, 7 Fabry and 10 ASMD) and 15 healthy samples were analyzed to determine whether or not the underlying disease could be identified with exclusion of false-positives and false-negatives.

## 5. Conclusions

This extension on the earlier proven accurate and robust detection method for the Lyso-biomarkers GlcSph, Lyso-Gb3 and Lyso-SM on DBS offers more clinical opportunities in diagnostics and follow-up of patients with Gaucher, Fabry and ASMD. The DBS is the primary chosen matrix for these analyzations due to its reduced costs and user-friendly experience and stability of lyso-biomarkers measured. Capitainers are proven to have a significant role in this laboratory work up as well since they excel specifically for Lyso-SM detections and eliminate the volume-related hesitations for measurements due to their filter-controlled blood volumes. Finally this method can yield great promise towards the implementation of early detection of sphingolipidoses in newborn screening.

## Figures and Tables

**Table 1:** Results for R^2^, Spearman’s p, significance for linearity and the regression equation obtained.

DBS	R^2^	Spearman’s p	Significant with	Slope	Regression Equation	Conclusion
**GlcSph**	0.977	0.988	<0.001	0.99	0.99x − 0.26	Good significant correlation
**Lyso-Gb3**	0.973	0.986	<0.001	0.99	0.99x − 0.02	Good significant correlation
**Lyso-SM 509**	0.963	0.981	<0.001	1	1x + 0.04	Good significant correlation
**CAP**	**R^2^**	**Spearman’s p**	**Significant with**	**Slope**	**Regression equation**	**Conclusion**
**GlcSph**	0.979	0.989	<0.001	1.03	1.03x − 0.11	Good significant correlation
**Lyso-Gb3**	0.978	0.989	<0.001	1.01	1.01x − 0.01	Good significant correlation
**Lyso-SM 509**	0.979	0.989	<0.001	1.01	1.01x − 0.13	Good significant correlation

**Table 2:** Precision.

GlcSph	QC Level	Precision, CV% Intra-Assay DBS	Precision, CV% Inter-Assay DBS	Precision, CV% Intra-Assay CAP	Precision, CV% Inter-Assay CAP
	QC 1	12.4	10	10	4.9
	QC 2	9.7	7.9	5.3	6.2
	QC 3	9.6	6.6	4.6	9.3
	QC 4	5.5	5	5.1	7
	QC 5	6.6	3.9	4.3	10.5
**Lyso-Gb3**	**QC level**	**Precision, CV%** **Intra-Assay DBS**	**Precision, CV%** **Inter-Assay DBS**	**Precision, CV%** **Intra-Assay CAP**	**Precision, CV%** **Inter-Assay CAP**
	QC 1	12.7	9.3	22	0
	QC 2	8	13.6	8.3	4.5
	QC 3	7.1	5.5	7.3	2.9
	QC 4	4.7	8	5.4	7.2
	QC 5	6	0	4.9	9.3
**Lyso-SM**	**QC level**	**Precision, CV%** **Intra-Assay DBS**	**Precision, CV%** **Inter-Assay DBS**	**Precision, CV%** **Intra-Assay CAP**	**Precision, CV%** **Inter-Assay CAP**
	QC 1	30.8	52.8	20.6	12.7
	QC 2	19.7	33.7	9.5	4.8
	QC 3	10.4	10.5	5.8	6.2
	QC 4	6	5.7	5.2	8.6
	QC 5	7.8	6.3	4.8	9.8

Measurements represented as the coefficient of variance percentage (CV%); QC: quality control; cut-off defined by the CLSI C62 and CLSI EP05 guidelines is 15%.

**Table 3:** Accuracy.

GlcSph	QC Level	Accuracy, RE% Inter-Assay DBS	Accuracy, RE% Inter-Assay CAP
	QC 1	0.5	3
	QC 2	13.2	6.9
	QC 3	−2	9.9
	QC 4	5.8	5.6
	QC 5	−4.5	5.4
**Lyso-Gb3**	**QC Level**	**Accuracy, RE%** **Inter-Assay DBS**	**Accuracy, RE%** **Inter-Assay CAP**
	QC 1	25	5.8
	QC 2	24.7	4.7
	QC 3	1.8	8.2
	QC 4	14.7	13.7
	QC 5	0.2	11.8
**Lyso-SM**	**QC Level**	**Accuracy, RE%** **Inter-Assay DBS**	**Accuracy, RE%** **Inter-Assay CAP**
	QC 1	−3.5	0.5
	QC 2	39.3	0.2
	QC 3	−5.2	2.8
	QC 4	3.3	6.4
	QC 5	−11	3.9

Measurements represented as the coefficient of variance percentage (CV%); QC: quality control; cut-off defined by the CLSI C62 and CLSI EP05 guidelines is 15%.

**Table 4:** Carry-over.

	Cut-Off	Low-Low Mean	High-Low Mean	Carry-Over Absolute	Conclusion
GlcSph	1.34	8.68	9.39	0.71	Below cut-off
Lyso-Gb3	2.65	2.74	3.033	0.29	Below cut-off
Lyso-SM	7.60	8.44	10.36	1.92	Below cut-off

The ‘low-low mean’ and ‘high-low mean’ columns represent the mean value of the low after low analysis and the mean value of the low concentrations measured after a high concentration, respectively. Results are expressed in ng/mL (or 10^−6^ g/L). The ‘carry-over absolute’ column represents the difference between the mean detected concentrations. The ‘cut-off’ column represents the cut-offs based on the CLSI guidelines.

**Table 5:** LOQ.

DBS	Cut-Off	Limit for LOQ	Obtained LOQ
GlcSph	320	3200	14,400
Lyso-Gb3	76	760	1560
Lyso-SM	490	4900	86,200
**CAP**	**Cut-Off**	**Limit for LOQ**	**Obtained LOQ**
GlcSph	600	6000	32,800
Lyso-Gb3	125	1250	30,750
Lyso-SM	330	3300	2,428,500

The cut-off represents the average noise in the measurements. Limit for LOQ is the minimum for achieving a good LOQ sufficient for an S/N ratio greater than 10 based on the determined cut-off. The obtained LOQ is the effective average measured LOQ value. All obtained values clearly meet the pre-imposed conditions.

**Table 6:** Case findings.

	GlcSph (ng/mL)	Lyso-Gb3 (ng/mL)	Lyso-SM (ng/mL)	Diagnosis
Patient sample 1	0.76	0.04	266.94	ASMD
Patient sample 2	0.93	73.16	37.1	Fabry
Patient sample 3	524.5	1.52	34.24	Gaucher
Patient sample 4	211	0.72	26.64	Gaucher
Patient sample 5	1.03	0.52	300.39	ASMD
Patient sample 6	3.94	0.11	205	ASMD
Patient sample 7	1.67	79.1	20.17	Fabry
Patient sample 8	286.65	1.53	0	Gaucher
Patient sample 9	1.12	1.04	400.37	ASMD
Patient sample 10	397.79	2.25	0	Gaucher
Patient sample 11	0.18	30.5	0.49	Fabry
Patient sample 12	0.4	22.97	6.11	Fabry
Patient sample 13	0.63	1.44	617.45	ASMD
Patient sample 14	1.23	10.9	18.96	Fabry
Patient sample 15	68.94	0.94	19.19	Gaucher
Patient sample 16	2.67	2.34	1367.31	ASMD
Patient sample 17	2	1.29	114.90	ASMD
Patient sample 18	0.4	22.97	6.11	Fabry
Patient sample 19	440	2.69	52.76	Gaucher
Patient sample 20	2.06	1.49	110.87	ASMD
Patient sample 21	166.89	1.55	44.00	Gaucher
Patient sample 22	1.09	7.55	3.86	Fabry
Patient sample 23	90.75	0.52	27.66	Gaucher
Patient sample 24	0.49	0.55	479.63	ASMD
Patient sample 25	78	0.25	54.39	Gaucher
Patient sample 26	1.12	1.04	400.37	ASMD
Patient sample 27	0.63	0.19	35.34	Healthy
Patient sample 28	3.30	0.42	24.78	Healthy
Patient sample 29	2.32	0.36	26.16	Healthy
Patient sample 30	1.88	0.16	33.11	Healthy
Patient sample 31	2,65	0.57	40.65	Healthy
Patient sample 32	1.12	0.08	30.27	Healthy
Patient sample 33	1.015	0.17	38.78	Healthy
Patient sample 34	2.022	0.20	16.13	Healthy
Patient sample 35	0.54	0.08	7.078	Healthy
Patient sample 36	3.45	0.42	14.87	Healthy
Patient sample 37	2.27	0.19	30.46	Healthy
Patient sample 38	1.66	0.41	40.93	Healthy
Patient sample 39	3.09	0.19	33.36	Healthy
Patient sample 40	0.86	0.51	45.90	Healthy
Patient sample 41	1.83	0.28	43.16	Healthy

Case finding results whereby the measurements of GlcSph, Lyso-Gb3 and Lyso-SM measured at once with the represented method are shown. Results indicated in a gray box are aberrant and positive patient samples for the corresponding disorder.

**Table 7:** Overview of the standard concentrations.

Standard (STD)	STD1	STD2	STD3	STD4	STD5	STD6	STD7
GlcSph (ng/mL)	1	5	10	20	100	500	1000
Lyso-Gb3 (ng/mL)	0.2	0.5	2	8	80	160	400
Lyso-SM (ng/mL)	1	5	10	20	100	500	1000

**Table 8:** Overview of the quality control concentrations.

Quality Concentration	QC1	QC2	QC3	QC4	QC5
GlcSph (ng/mL)	4	10	40	200	800
Lyso-Gb3 (ng/mL)	1.2	3	10	50	200
Lyso-SM (ng/mL)	4	10	40	200	800

**Table 9:** QTRAP5500 and LC system settings.

Compound Parameters							
MRM	Test	Dwell Time (ms)		DP (Volts)	CE (Volts)		CXP (Volts)
**1**	GlcSph-IS	30.0		176.0	31.0		20.0
2	GlcSph	30.0		176.0	31.0		20.0
3	Lyso-Gb3-IS	60.0		171.0	45.0		20.0
4	Lyso-Gb3	60.0		171.0	45.0		20.0
5	Lyso-SM d7	30.0		176.0	31.0		20.0
6	Lyso-SM	30.0		176.0	31.0		20.0
**LC-MS/MS source settings**							
**Total Flow**	**Pressure Limits**	**Needle Stroke**	**Sampling Speed**	**Cooler Temp**	**Oven Temp**	**ESI Needle**	**Rinsing Volume**
0.50 mL/min	14,000 psi	50 mm	2.0 µL/sec	15 °C	40 °C	50 mm	500 µL
**EP**	**CUR**	**CAD**	IS*	**TEM**	**GS1**	**GS2**	
10 V	35 psi	medium	5500 V	600 °C	60 psi	50 psi	

CE, capillary electrophoresis; CUR, curtain gas; CXP, collision cell exit potential; DP, declustering potential; EP, entrance potential; ESI, electrospray ionization; GS1, ion source gas 1; GS2, ion source gas 2; IS, internal standard; IS*, ionspray voltage; LC-MS/MS, liquid chromatography with tandem mass spectrometry; MRM, multiple reaction monitoring; ms, milliseconds; psi, pound-force per square inch; TEM, temperature; and V, volts.

**Table 10:** Summary of the preparation steps for the detection method.

Working Solution	Extraction Method
50% MeOH	30 min sonication at 30 °C
+50% DMSO	+20 min shaker at 37 °C
+30 µL IS on 9.07ml	+10 min centrifuging at 4750 rpm

MeOH, methanol; DMSO, dimethylsulfoxide2; and IS, combination of internal standards ^13^C_6_-Lyso-Gb3, ^13^C_6_-Lyso-Gb1 and Lyso-SM-d7.

## Data Availability

Data is contained within the article.

## List of Abbreviations

LC-MS/MS	Liquid chromatography with tandem mass spectrometry
CLSI	Clinical and Laboratory Standards Institute
LSD	Lysosomal storage diseases
GD	Gaucher disease
FD	Fabry disease
ASMD	Acid sphingomyelin deficiency
Gb1	Glycosphingolipid glucosylceramide
GlcSph	Lyso-glucosylsphingosine
Gb3	Globotriaosylceramide
Lyso-Gb3	Globotriaosylsphingosine
Lyso-SM	Lyso-sphingomyelin
ERT	Enzyme replacement therapy
SRT	Substrate reduction therapy
DNA	Desoxyribonucleic acid
CCL18	C-C motif chemokine ligand 18
DBS	Dried blood spot
QC	Quality control
DMSO	Dimethylsulfoxide
MeOH	Methanol
RBCs	Red blood cells
K3-EDTA	K3 potassium salt of ethylene diamine tetra acetic acid
WBCs	White blood cells
ESI	Electrospray ionization
MRM	Multiple reaction monitoring
IS	Internal standard
LOQ	Limit of quantification
CV%	Coefficient of variance
SD	Standard deviation
S/N	Signal-to-noise ratio

## List of Human Genes

GBA1	Glucosylceramidase beta 1, HGNC ID 4177
Alias symbols	GBA, GCB, GLUC, alias names: glucocerebrosidase
GLA	Galactosidase alpha, HGNC ID 4296
Alias symbols	GALA
SMPD1	Sphingomyelin phosphodiesterase 1, HGNC:11120, OMIM 607616
Alias symbols	ASM alias names: acid sphingomyelinase, Niemann–Pick type A/B
